# LASIK Complications and the Internet: Is the Public being Mislead?

**DOI:** 10.2196/jmir.5.1.e2

**Published:** 2003-03-31

**Authors:** Daragh Kennedy Fahey, Julius Weinberg

**Affiliations:** ^1^City UniversityMIM CentreLondonEngland; ^2^City UniversityInstitute of Health SciencesLondonEngland

**Keywords:** Keratomileusis, laser in situ, LASIK, postoperative complications, Internet, public

## Abstract

**Background:**

LASIK (Laser in Situ Keratomileusis) is a very popular combined surgical and laser procedure, which is used to correct myopia (shortsightedness) and hyperopia (farsightedness). There is concern that the public is being misled regarding the safety of the procedure. very popular combined surgical and laser procedure, which is used to correct myopia (shortsightedness) and hyperopia (farsightedness). There is concern that the public is being misled regarding the safety of the procedure.

**Objectives:**

To assess the quality and quantity of the information on complications on LASIK Web sites. information on complications on LASIK Web sites.

**Method:**

Serial analysis and evaluation of the authorship, content, and technical quality of the information on the complications of LASIK on 21 Web sites. content, and technical quality of the information on the complications of LASIK on 21 Web sites.

**Results:**

Of the 21 LASIK Web sites visited, 17 were commercial. Of the 21 Web sites, 5 (24%) had no information on complications. Of the 16 sites that had information on complications the author of the information was clearly identified in 5 (31%), the content was only referenced in 2 (12.5%), and evidence of the information having been updated was only seen in 2 (12.5%). The quantity of information is generally minimal and the information itself is generally difficult to understand and locate. commercial. Of the 21 Web sites, 5 (24%) had no information on complications. Of the 16 sites that had information on complications the author of the information was clearly identified in 5 (31%), the content was only referenced in 2 (12.5%), and evidence of the information having been updated was only seen in 2 (12.5%). The quantity of information is generally minimal and the information itself is generally difficult to understand and locate.

**Conclusions:**

The quality and quantity of the information on the Web on the complications of LASIK are poor. More work is required to encourage clear, accurate, up-to-date, clearly authored, and well-referenced, balanced ophthalmic information. on the Web on the complications of LASIK are poor. More work is required to encourage clear, accurate, up-to-date, clearly authored, and well-referenced, balanced ophthalmic information.

## Introduction

The Internet has become increasingly popular among consumers as a source of health care information. A poll in 2001 showed that almost 100 million American adults regularly go online for health care information [1].

Although there are quality-standards guidelines for medical publishing on the Internet [2,3] there is currently no governing body acting as a gatekeeper of Web page publications. Anybody can create a Web site and they are free to write whatever they wish. This has led to some serious concern as to the accuracy of the information of some of these sites [4,5]. Even those sites that offer to evaluate health Web sites are often incomplete or fail to reveal how they perform the evaluation [6,7].

LASIK (Laser in Situ Keratomileusis) is a very popular [8] combined surgical and laser procedure, which is used to correct myopia (shortsightedness) and hyperopia (farsightedness). As the procedure is not available within the United Kingdom's National Health System, but is available privately, many commercial companies have taken a keen interest in promoting its uptake. Although LASIK is predominantly a safe procedure there are many potential complications [9- 16]. Most of these are mild and/or transient but some are severe, permanent, and may require corneal grafts to correct [10,11]. LASIK was first performed only 11 years ago and United States Food and Drug Administration approval was only granted 6 years ago; therefore there is concern as to what may happen to these corneas in the future.

Based on a Medline search there have been no previous studies that have specifically examined the quality of ophthalmic Web sites. This study examines information a member of the public might read when searching for information on LASIK on the Internet. The sites are evaluated regarding the quality of the content, authorship, and technical aspects of the information on the complications of LASIK. that have specifically examined the quality of ophthalmic Web sites. This study examines information a member of the public might read when searching for information on LASIK on the Internet. The sites are evaluated regarding the quality of the content, authorship, and technical aspects of the information on the complications of LASIK.

## Methods

Between July 16 and July 22, 2002, 21 Web sites that described LASIK were evaluated. Included in the evaluation were the first 17 English-language sites that appeared when *LASIK* was searched using the Google search engine [17] and the first 4 UK (United Kingdom) sites selected using the same keyword but using the Yahoo [18] search engine and limiting the selection to UK sites. Although the Google search returned 150000 hits and the Yahoo search returned 1010 hits, the evaluation was restricted to the first 21 English-language sites that appeared, as it was felt that most English-speaking potential LASIK customers would not extend their search beyond this number of sites. There was no consumer involvement when devising the search strategy. Of the Web sites included in the study, 16 were North American, 4 were United Kingdom, and 1 was Indian. Web sites that just provided information on addresses of LASIK surgeons/surgeries, were not included.

Each site was assessed by one rater (DF) who was blinded to the source. Objective measurement was performed of the following: source. Objective measurement was performed of the following:

- Whether the Web site was commercial, academic, government, or by an individual. an individual.
- Where the site was from (ie, United States, United Kingdom, or elsewhere). elsewhere).
- Whether the site dealt solely with LASIK, with different types of eye laser procedures, or with various eye conditions. of eye laser procedures, or with various eye conditions.
- Whether information on complications was given.
- Whether a "last-updated" record of the page with the information on complications was given."last-updated" record of the page with the information on complications was given.
- Whether the author of the information was identified.
- Whether links (ie, to the explanation of medical terms) or relevant graphics were used to explain the information. relevant graphics were used to explain the information.
- Whether responsibility for the information given is waivered. waivered.
- Whether a consent form is available online.

Most of the information on complications was assessed and marked subjectively based on authorship, content, and technical quality. The evaluation form used (Table 1) for the Web sites visited was created based on appraisal criteria from a number of sources [2,4,5,19- 21].

**Table 1:** Evaluation form used for LASIK Web sites

**Category**	**Mean Score(maximum = 10)**
*Authorship*	
Recognized authority	
Credentials/Experience	
Contact information	
*Content*	
Details of complications	
Easy to understand	
Ease of locating complications	
Accuracy of references*	
Up-to-date information*	
Balanced information*	
*Technical*	
Quality of referenced page's header, body and footer	
Ease of identifying Web site's headings and subheadings	

^*^ Only applies to 2 websites.

### Evaluation form details

#### Authorship of Information on Complications

##### Recognised Authority

This score was based on whether and to what degree the author is a recognized authority in the area. The sites scored higher if the authors were ophthalmologists, if they perform many LASIK procedures themselves, and if they have publications in the area. a recognized authority in the area. The sites scored higher if the authors were ophthalmologists, if they perform many LASIK procedures themselves, and if they have publications in the area.

##### Credentials/Experience

This score was based on whether and to what extent the author's experience and credentials were provided. The more information, given the higher the score. experience and credentials were provided. The more information, given the higher the score.

##### Contact Information

This score was based on the amount of contact information that was given to get in touch with the author for further enquiries. Full marks were given if a telephone number, e-mail address, and postal address were provided. was given to get in touch with the author for further enquiries. Full marks were given if a telephone number, e-mail address, and postal address were provided.

#### Content

##### Details of Complications

This score was based on how much was written on complications. The more complications that were mentioned, and the more information (such as frequency, clinical course, and treatment options) that was given on each complication, the higher the mark. The more complications that were mentioned, and the more information (such as frequency, clinical course, and treatment options) that was given on each complication, the higher the mark.

##### Easy to understand

This score was based on how easy it would be for a member of the public to understand the information on complications. The highest marks were given when the authors felt the complications were well explained with diagrams and/or video and when medical jargon was either not used or well explained (either directly or by using hyperlinks). The information given was also analyzed objectively using the Flesch-Kincaid reading scale and Flesch reading ease score. public to understand the information on complications. The highest marks were given when the authors felt the complications were well explained with diagrams and/or video and when medical jargon was either not used or well explained (either directly or by using hyperlinks). The information given was also analyzed objectively using the Flesch-Kincaid reading scale and Flesch reading ease score.

##### Ease of Locating Complications

This score was based on how easily a member of the public could locate information on complications from the home page of the Web site. High scores were given when there was an obvious link to the complications from the home page, when the information on complications came under the heading *Complications*, and if there was a search engine on the site that could target the complication information if the words *risk* or *complications* were input.

##### Accuracy of References

This score was based on the accuracy of the references given on the information on complications. All references were read (full text) and the highest scores were given if the referenced information was to be found there and if it had been quoted in context. the information on complications. All references were read (full text) and the highest scores were given if the referenced information was to be found there and if it had been quoted in context.

##### Up-to-date Information

This score was based on the degree to which the information was up-to-date. Highest scores were given for information from the most recent studies. up-to-date. Highest scores were given for information from the most recent studies.

##### Balanced Information

This score was based on the proportion of, and the degree to which, the information was balanced. Highest scores were given for impartial information that wasn't overly optimistic or pessimistic. which, the information was balanced. Highest scores were given for impartial information that wasn't overly optimistic or pessimistic.

#### Technical Quality

##### Quality of Referenced Page's Title, Body, and Footer Footer

This score was based on the quality of the page with information on complications. Highest marks were given if the title, body of text, and footer were easy to identify and if they provided relevant information. on complications. Highest marks were given if the title, body of text, and footer were easy to identify and if they provided relevant information.

##### Ease of Identifying Web Site Headings and Subheadings Subheadings

This score was based on the quality of the Web sites headings and subheadings. Highest scores were provided when the Web site had clear intuitive headings and subheadings that the authors felt would aid the visitor's navigation through the Web site. and subheadings. Highest scores were provided when the Web site had clear intuitive headings and subheadings that the authors felt would aid the visitor's navigation through the Web site.

##### Statistics

Nominal data was expressed as percentages. Sample proportions were compared by hypothesis testing with a 5% significance level using the MINITAB statistical software package. Subjective scores for the Web sites were expressed as a mean and standard deviation, which also was calculated using MINITAB. were compared by hypothesis testing with a 5% significance level using the MINITAB statistical software package. Subjective scores for the Web sites were expressed as a mean and standard deviation, which also was calculated using MINITAB.

## Results

**Figure 1:**
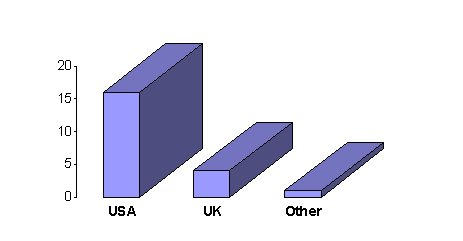
Origin of Web sites visited (out of a total of 21)

**Figure 2:**
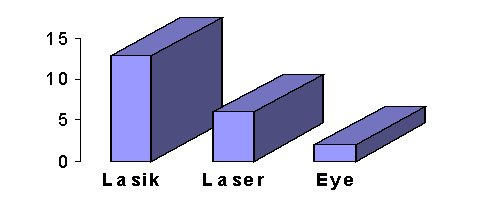
Type of Web site visited (out of a total of 21)

General information about the Web sites visited is summarized in Figure 1 and Figure 2. 16 of the 21 sites visited were from the United States and 13 of the sites were devoted to LASIK only.

**Figure 3:**
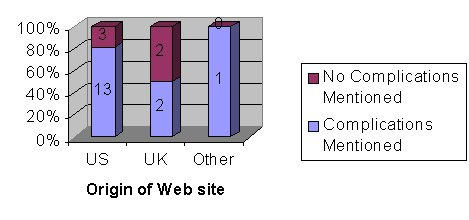
Number and percentage of Web sites (out of a total of 21) that mentioned complications

**Figure 4:**
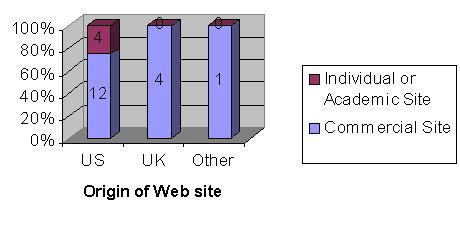
Number and percentage of Web sites (out of a total of 21) that were commercial

Figure 3 shows that 16 of the 21 the sites mentioned the complications of LASIK and this was more common in the American sites compared with UK sites (75% vs 50%), although only 4 of the sites visited were UK. This is not a statistically significant difference ( *P*= .285, 95% CI). Figure 4 illustrates that 12 out of the 16 US (United States) sites visited were commercial compared with all 4 of the UK sites. This is not a statistically significant difference ( *P*= .264, 95% CI). Twelve out of the 16 commercial sites compared with 4 out of the 5 individual/government sites gave information on complications of LASIK, which is also not a statistically significant difference ( *P*= .819, 95% CI).

**Table 2:** Number of the 16 Web sites with information on complications that had certain types of complication information

**Complication Information**	**Number**
Last updated	2
Authorship identity	3
Referenced	6
Responsibility waivered?	6
Use of links	2
Use of relevant graphics	5
Consent form available online	1

Table 2 shows that out of the 16 sites that had details on complications, 2 showed when they were last updated, authorship of page could only be clearly identified in 3, and referenced information was only found in 6. Of the 16 sites visited only 2 used links to enhance the quality of their complication information and only 6 waivered any responsibility for the information given.

Figure 3 showed that 5 of the 21 Web sites visited had no information on the possible complications of LASIK. In those sites that have information on complications, Table 2 and Table 3 illustrate that it is often inadequate because of:

- Poorly identified and/or inappropriate authorship.
- Poor contact information for author.
- Incomplete.
- Difficult to locate.
- Poorly referenced content.
- Difficult to understand.
- Explanation not enhanced with graphics and/or hyperlinks.
- Not up-to-date.
- Imbalance.

Table 3 shows that of the 2 Web sites that provided information on the complications the references were generally accurate (score 8 out of 10) and were mostly were up-to-date (score 6 out of 10). One of these Web sites scored 2 out of 10 for balanced information because there was only 1 quoted reference for all its information; this was from a commercial site.

Although this study presents a mostly-negative picture of the complication information on LASIK Web sites there were some positive findings. Table 3 shows that most sites scored well on the quality of headings, subheadings and footers.

**Table 3:** Scores out of 10 of the criteria used to evaluate each Web site

**Category**	**Mean Score(Maximum = 10)**
Authorship	
Recognized authority	5.4 (SD= 2.3)
Credentials/Experience	6.6 (SD= 3.3)
Contact information	4.5 (SD= 4.5)
Content	
Details of complications	3.1 (SD= 1.8)
Easy to understand	4.6 (SD= 1.5)
Ease of locating complications	4.8 (SD= 2.5)
Accuracy of references*	8 (SD= 0)
Up-to-date information*	6 (SD= 0)
Balanced information*	4 (SD= 2.8)
Technical	
Quality of referenced page's header, body and footer	6.8 (SD= 1.9)
Ease of identifying Web site's headings and subheadings	7.3 (SD= 1.8)

^*^ Only applies to 2 websites.

## Discussion

This study assessed the quality of information on the Internet on LASIK complications and found it to be poor. Our findings support recent work in the College of Optometry in Southern California [22], which looked at 96 Web sites containing LASIK information. They rated 26% of sites as "markedly informative," 28% were rated "moderately informative," and 46% were rated as "minimally informative." The poor quality of the information represents a negligent omission as the public are being misled into believing that LASIK is without risk. This may lead to liability cases by patients with complications whose decision to have LASIK was based on the information they read on the Web site. Of the sites that gave information on the complications of LASIK, only 38% (Table 2) included a waiver of responsibility for the information. There is an argument that the authors of the Web site could be held liable unless they have a waiver. On the other hand perhaps health Web sites should be prevented from using a waiver to ensure greater accuracy of the information provided.

Detailed, well-referenced, up-to-date, good quality information from recognized authorities should be found on all medical Web sites. This is particularly important for procedures such as LASIK that are often primarily cosmetic but whose complications can be devastating. The risk of irreparable sight-threatening complications such as corneal ectasia [11] particularly needs to be explained. The combination of graphics, video, and hyperlinks should be fully utilized to explain difficult concepts and allow patients to make fully-informed decisions regarding their medical treatment. Comprehensive consent forms available on the Internet should be mandatory for elective procedures such as LASIK. This was only seen in 1 of the Web sites analyzed [23].

In a systematic review of studies that assess the quality of health information for consumers on the World Wide Web, Eysenbach et al [24] conclude that "the individual's risk of encountering an inadequate site on the Web is a function of both the proportion inadequate information on the Web (P) and the inability (I) of the individual (or his tools) to filter the inadequate sites." Because of the large public interest in LASIK it is the opinion of the authors that all LASIK Web sites should adhere to strict standards regarding what is published. The public is more likely to trust information from a government/educational site, but all the UK sites located in our study were commercial. It suggests that the UK government and/or ophthalmology institutions need to produce a good quality Web site on LASIK for the public to use.

Deciding on the most appropriate method used to control/guide health care information on the Web is difficult. Current methods include filtering tools, quality labels, codes of conduct, and user-guidance labels [25]. The National Electronic Library for Health uses "methodical, organized human reviewing, selection and filtering based on well-defined quality appraisal criteria" for their site [26]. There is a subjective bias in this system and one might argue that filtering systems violate human rights by acting as a censor of health information. The eHealth Code of Ethics of the Internet Health Coalition [27] is an example of a code of conduct. The coalition is an organization that has developed a set of quality criteria for those wishing to produce health-related Web sites. If the public knows that a Web site adheres to a certain standard when it was produced then one could avoid misleading Web sites that adopt misleading tactics such as picking up the client machine's current date and displaying it after the "last updated" remark, thus giving a false impression of the currency of the information [26]. Net Scoring [28] is an example of a user guidance system that enables users to check if a site and its contents comply with certain standards by accessing a series of questions from a displayed logo. This requires a considerable amount of effort on the part of the user. It is the opinion of the authors that the best solution is to have accredited third-party approval "stamps" that would be clearly visible on the Web sites. For example, the LASIK Web sites might have a comment after the description of the complications that says "This information has been reviewed and approved by the Royal College of Ophthalmologists, UK." Selection of a correct approach is controversial and some authors such as Delamothe [29] argue that "rating the quality of medical Web sites may be impossible *"* and that "one option is to rate the process by which the content was produced rather than the content itself - a medical journal's Web site containing peer-reviewed material would rate higher than a commercial site selling miracle cures for cancer."

The results of this study are somewhat limited in that part of the evaluation is subjective; however, this is balanced with some important objective assessments (such as the number of sites which mention complications). the evaluation is subjective; however, this is balanced with some important objective assessments (such as the number of sites which mention complications).

Many LASIK Web sites are giving minimal, dated, poor-quality, and inaccurate information on its complications and this can mislead the public. In many cases there is no authorship of the information and in those cases where authorship is clear the information may not come from a recognized authority. Authors should publish information that is easy to understand and locate. The explanations of the complications should be supplemented by the excellent multimedia capacity of Web sites with many images and links. One solution is for authorities such as the Royal College of Ophthalmologists to evaluate UK ophthalmic sites and offer to provide a stamp of approval for those that fit their good-quality criteria. We conclude that the quality of information on the Internet on the complications of LASIK is poor and needs to be addressed. As Wyatt [4] says, "unless we evaluate the quality of clinical sites and their effects on users, we risk drowning in a sea of poor quality information."

## Abbreviations

UK: United Kingdom
US: United States
